# Appropriateness of antibiotic treatment in intravenous drug users, a retrospective analysis

**DOI:** 10.1186/1471-2334-8-42

**Published:** 2008-04-03

**Authors:** Dominik Mertz, Nina Viktorin, Marcel Wolbers, Gerd Laifer, Bernd Leimenstoll, Ursula Fluckiger, Manuel Battegay

**Affiliations:** 1 Division of Infectious Diseases & Hospital Epidemiology, University Hospital Basel, Switzerland; 2 Basel Institute for Clinical Epidemiology, University Hospital Basel, Switzerland; 3 Division of Internal Medicine, University Hospital Basel, Switzerland

## Abstract

**Background:**

Infectious disease is often the reason for intravenous drug users being seen in a clinical setting. The objective of this study was to evaluate the appropriateness of treatment and outcomes for this patient population in a hospital setting.

**Methods:**

Retrospective study of all intravenous drug users hospitalized for treatment of infectious diseases and seen by infectious diseases specialists 1/2001–12/2006 at a university hospital. Treatment was administered according to guidelines when possible or to alternative treatment program in case of patients for whom adherence to standard protocols was not possible. Outcomes were defined with respect to appropriateness of treatment, hospital readmission, relapse and mortality rates. For statistical analysis adjustment for multiple hospitalizations of individual patients was made by using a generalized estimating equation.

**Results:**

The total number of hospitalizations for infectious diseases was 344 among 216 intravenous drug users. Skin and soft tissue infections (n = 129, 37.5% of hospitalizations), pneumonia (n = 75, 21.8%) and endocarditis (n = 54, 15.7%) were most prevalent. Multiple infections were present in 25%. Treatment was according to standard guidelines for 78.5%, according to an alternative recommended program for 11.3%, and not according to guidelines or by the infectious diseases specialist advice for 10.2% of hospitalizations. Psychiatric disorders had a significant negative impact on compliance (compliance problems in 19.8% of hospitalizations) in multiple logistic regression analysis (OR = 2.4, CI 1.1–5.1, p = 0.03). The overall readmission rate and relapse rate within 30 days was 13.7% and 3.8%, respectively. Both non-compliant patient behavior (OR = 3.7, CI 1.3–10.8, p = 0.02) and non-adherence to treatment guidelines (OR = 3.3, CI 1.1–9.7, p = 0.03) were associated with a significant increase in the relapse rate in univariate analysis. In 590 person-years of follow-up, 24.6% of the patients died: 6.4% died during hospitalization (1.2% infection-related) and 13.6% of patients died after discharge.

**Conclusion:**

Appropriate antibiotic therapy according to standard guidelines in hospitalized intravenous drug users is generally practicable and successful. In a minority alternative treatments may be indicated, although associated with a higher risk of relapse.

## Background

Infectious diseases are a major cause of morbidity and mortality among intravenous drug users (IVDU) [1-8]. Malnutrition, immunodeficiency, homelessness, and needle-sharing contribute to a high infection rate in these patients [7,9]. Thus, reducing these risk factors and providing adequate medical care are important aims of opioid maintenance programs [10,11]. In general, IVDU seek medical treatment in emergency departments more often and also need to be hospitalized more often than patients in an age-matched non-IVDU population [4,10]. They also tend to seek medical attention late in the course of a disease [4]. The resulting delay in diagnosis and possibly reduced number of available therapeutic options may produce a less favorable outcome to treatment as well as more frequent and potentially life-threatening complications.

Non-adherence to treatment protocols is a frequent and persistent problem in hospitalized IVDU [12]. Therefore, there may be a perception among health care workers that IVDU will be less willing to consent to or follow a specific antibiotic therapy. Such a perception might lead them to prescribe a less than optimum treatment program because the preferred protocol would be difficult for the patient to follow correctly.

Psychiatric co-morbidities in IVDU are reported in up to 30% of IVDU and are recognized as risk factors for needle-sharing, more frequent sex for money or gifts and being raped [13], all of which can lead to infection. Patients with psychiatric diseases are known to have a limited capacity to consent to proposed interventions and are consequently more likely to refuse treatment [14]. Psychiatric disease as well as intravenous drug use are known risk factors for patients leaving the hospital against medical advice and for not adhering to treatment protocols [15].

Data is still scarce about success rates for treatment of infectious disease in IVDU in a hospital setting, including the extent of non-compliant patient behavior and its impact on the efficacy of treatment and patient outcomes. Therefore, the goal of the present study was to analyze the appropriateness of treatment protocols, compliance with treatment by patients, and the outcomes of therapy in IVDU who were hospitalized with infectious diseases and were seen by a specialist in infectious diseases. The hypothesis was that the appropriateness of treatment protocols in IVDU was higher than suspected by most physicians and that the outcome of treatment depends on the appropriateness of treatment and the compliance of the patients.

## Methods

### Study design

This was a retrospective study of all IVDU who were hospitalized for treatment of infectious diseases and were evaluated on demand by the infectious disease service over six years, January 2001 through December 2006, in the University Hospital Basel in Switzerland, a 780-bed primary and tertiary care center with approximately 27,000 admissions annually.

One single investigator extracted the following data from hospital charts as well as from the separate medical charts of the infectious disease specialists who were consulted: Name; gender; date of birth; date of admission; duration of hospitalization; date of infectious disease specialist consultations; duration of illicit drug use and which substances used; participation in an opioid maintenance program; main diagnosis and co morbidities; body mass index; infectious diseases diagnosis; main pathogen; additional pathogens; antibiotics, doses, application forms and durations of treatment; complications (i.e. acute renal failure, adult respiratory distress syndrome, disseminated intravasal coagulation, septic shock); admission to intensive care unit (ICU) and days on ICU; fatalities and reasons of death; fever, c-reactive protein and leucocytes at time of admission and time of discharge; time to defeverescence; readmission and reason for readmission. The data was entered in a database (SPSS 14, SPSS Inc., Chicago US), controlled by a second investigator and checked for plausibility during analysis of the dataset by another two independent reviewers.

Treatment protocols were evaluated with respect to their appropriateness for treating the diagnosed infection. Where treatment programs deviated from accepted standards, the reason for the use of an alternative program was noted. Participation in any program of heroin or methadone substitution administered by a public organization was considered participation in an opioid maintenance program.

A patient's behavior was considered to be non-compliant if it was disruptive and violated hospital rules according to the charts, i.e., if the patient did not comply with diagnostic measures or adhere to therapeutic measures, left the hospital against medical advice, continued intravenous drug use during hospitalization, smoked in the room, or assaulted hospital staff.

The study was approved by the Ethics Committee of the Cantons Basel-Stadt and Basel-Land (EKBB).

### Outcome parameters

The outcome parameters were the appropriateness of the prescribed therapy (the choice of treatment and the duration of treatment with respect to standard guidelines for treating specific infectious diseases), the readmission rate (overall rate and the rate due to relapse), in-hospital mortality (both infectious-disease related and non-infectious-disease related), and outpatient mortality (death registries for the Canton Basel-Stadt).

Relapse was defined as infection by the same microorganism(s) responsible for the initial infection at the same site within 30 days after the patient was discharged from the hospital. For osteomyelitis, the time limit for relapse was extended to twelve months.

### Standards of diagnosis

Briefly, the most important definitions were: skin and soft tissue infections according to the practice guidelines of the Infectious Diseases Society of America [16]; pneumonia according to the European guidelines [17]; endocarditis according to the Dukes criteria [18]; septic thrombosis was defined by positive blood cultures and the detection of a thrombus in imaging [19,20], Finally, sepsis was defined by an infection and two or more SIRS (systemic inflammatory response syndrome) criteria [21]. Other diagnoses of infectious diseases were according to the Centers for Disease Control and Prevention (CDC) guidelines [22].

Major psychiatric disease, defined as major depression, schizophrenia, or personality disorders, was noted if mentioned in the final medical report as assed by a board certified psychiatrist.

Complications were defined as non-compliant patient behavior, renal failure, septic shock, or admission to the intensive care unit (ICU) during hospitalization.

### Standards of treatment

The appropriateness of the prescribed therapy was evaluated according to written internal guidelines of the infectious diseases division, which are closely adapted from international guidelines (The Sanford Guide To Antimicrobial Therapy 2007, [16,17,23,24]). Antibiotic therapy for septic thrombosis is based on expert opinions [23]. In our study, therapy for septic thrombosis was defined as adequate if a minimum duration of four weeks of treatment was planned and the antibiotic was given intravenously for the first 2 weeks [20] according to the guidelines for treating right heart endocarditis [24,25].

The appropriateness of treatment was analyzed by at least two investigators, which were not informed about the outcome of the cases at time of analysis. If there was no agreement, a third investigator was consulted. Whenever a pathogen was isolated, antibiotic susceptibility testing was considered for treatment. Our laboratories are working according to Clinical and Laboratory Standards Institute (CLSI) guidelines.

### Statistical analysis

A multiple logistic regression model was used to describe the dependence of patient compliance and in-hospital mortality on covariates, which were chosen according to clinical concerns. Adjustment for multiple hospitalizations of individual patients was made by using a generalized estimating equation (GEE) approach. Parameters were first estimated by using ordinary logistic regression, ignoring correlations between different hospitalization periods for a single patient (under "working independence"). Standard errors were then adjusted for repeat hospitalizations by using a robust sandwich estimator. The same statistical approach was used for univariate analyses. Results are given as odds ratio (OR) and 95% confidence interval (CI).

## Results

### Study population

**Figure 1:**
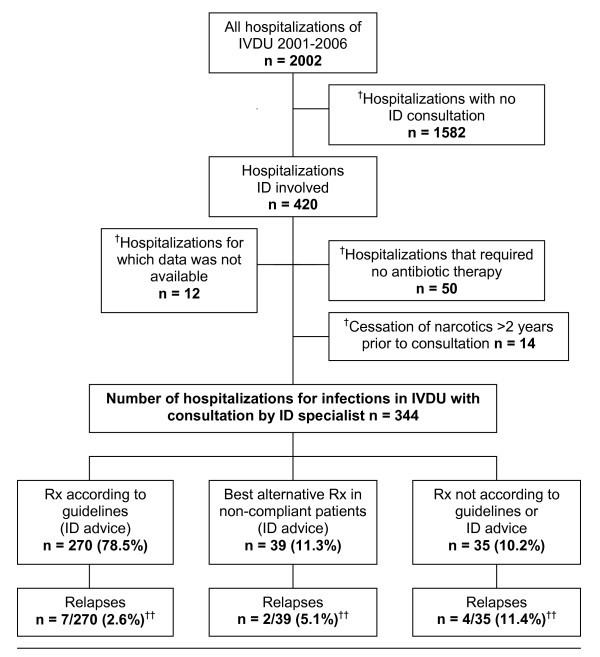
Study population and frequency of relapses according to different treatment modalities in 344 hospitalizations among 216 IVDU*. IVDU = Intravenous drug users. n = Number of hospitalizations. ID = Infectious disease specialist. Rx = Prescribed treatment. * seen by an ID during a period of 5 years. ^† ^Not included in study: -Patients who did not require antibiotic. -Former IVDU who had stopped using narcotics at least two years prior to their admittance to the hospital and were not participating in an opioid maintenance program ^††^p = 0.03 (Rx according to guidelines vs. others)

During six years, between January 2001 and December 2006, there were 2002 hospitalizations of IVDU (Figure 1). In 420 of these hospitalizations, a specialist in infectious diseases was consulted. Of these 420 cases, 76 (18.1%) were excluded from the study. Demographic data and laboratory results for the 344 hospitalizations of 216 patients are summarized in Table 1. Although the majority of IVDU (93.7%) were enrolled in an opioid maintenance program, 98.4% also used illicit drugs in addition to their maintenance doses. The patients in this study population suffered from diverse co-morbidities and malnutrition as indicated by the rather low body mass index.

**Table 1:** Patient characteristics of 344 hospitalizations among 216 IVDU*

			**%**
Gender (male/female) (n = 344)			67/33
Participation in opioid maintenance program (information available in n = 270)			
In a program = 253			93.7
No program = 17			6.3

		**median**	**(range)**
Age (years)	(n = 344)	38	(18–58)
Body mass index (kg/m^2^)	(n = 212)	20.9	(14–40)
Temperature at admission (°C)	(n = 328)	38.6	(35.3–42)
C-reactive protein at admission (mg/l)	(n = 342)	121.5	(0.7–514)
White blood cell count (× 10^9^/l)	(n = 342)	10.9	(0.1–80.4)
Length of hospital stay (days)	(n = 344)	17	(1–125)

^*^ seen by an ID during a period of 5 years.

^†^Data on opioid substitution program participation, body mass index, temperature, white cell count, and C-reactive protein was not available for all patients.

IVDU = Intravenous drug user.

n = Total number of hospitalizations for which specific data was available. (Total number of hospitalizations in this study = 344.).

ID = Infectious disease specialist

The median time to the first consultation by an infectious diseases specialist was 2 days (range 0–92 days). In median, two consultations (range 1–10) were performed per patient.

### Diseases and pathogens

The distribution of infectious diseases that were diagnosed in this population, the number of infections per hospitalization of a patient, the number of surgical interventions, and the comorbidities that were observed in the patients are summarized in Table 2. The most frequent diagnoses were skin and soft tissue infections followed by pneumonia and endocarditis. A high number of septic thromboses was diagnosed (n = 36, 10.5%). In addition, 23 non-septic thromboses of the deep venous system were diagnosed. Sepsis or severe sepsis due to different infections was documented in 35.5% (n = 122) cases, with no obvious clinical focus in 7.6% (n = 26). Of the 'various infections' listed in Table 1 (n = 75, 21.8%), the most frequently encountered were upper respiratory tract infections (n = 12), infections by Candida species (n = 10), urinary tract infections (n = 10), abdominal infections (n = 6), tuberculosis (n = 5) and sinusitis (n = 4). Eighty-six (25%) of the cases were characterized by two or more simultaneous infections. Viral co-infections, i.e., hepatitis C, HIV, and chronic hepatitis B, were common as comorbidities. Like IVDU in an earlier study [11,26], close to 80% of the patients in this study were positive for hepatitis C.

**Table 2:** Infectious disease diagnoses and co-morbidities in 344 hospitalizations among 216 IVDU*

	**n (%)**
Types of infectious disease diagnoses^†^:	
Skin and soft tissue infection	129 (37.5)
Pneumonia	75 (21.8)
Endocarditis	54 (15.7)
Bone and joint infections	50 (14.5)
Septic thrombosis	36 (10.5)
Primary sepsis without focus	26 (7.6)
Various infections	75 (21.8)
Number of simultaneous infections:	
one infection	258 (75.0)
two infections	70 (20.3)
three or more infections	16 (4.7)
Number of hospitalizations where surgical intervention was required	110 (32.0)

Comorbidities^†^:	
Major psychiatric disease	52 (15.1)
Alcohol addiction	55 (16.0)
Nicotin addiction	265 (77.0)
HIV infection	120 (34.9)
Chronic hepatitis B	47 (13.7)
Hepatitis C	268 (77.9)
Anemia	157 (45.5)
Deep venous thrombosis (non-septic)	23 (6.7)

IVDU = Intravenous drug users.

* seen by an ID during a period of 5 years.

^† ^447 diagnoses among 344 hospital episodes. During one hospital episode more than one diagnosis and comorbidity can be documented

Overall, a pathogen could be isolated in 78.8% of the cases (Table 3). The percentage of microbiologically documented infections was 100% for bacterial endocarditis and septic thrombosis, 96% for bone and joint infections, 85.7% for primary sepsis without focus, 77.7% for skin and soft tissue infections, and 72% for pneumonia. The percentage of microbiologically documented infections for all other types of infections together was 62.3%.

**Table 3:** Pathogens identified and antibiotic therapies used in 344 hospitalizations among 216 IVDU*

	**n (%)**
**Pathogens**	
*Staphylococcus aureus*	131^‡ ^(38.1)
Beta-hemolytic streptococci	42 (12.2)
Viridans streptococci	30 (8.7)
Enterobacteriaceae	17 (4.9)
*Streptococcus pneumoniae*	14 (4.1)
*Pseudomonas aeruginosa*	10 (2.9)
Coagulase-negative staphylococci	6 (1.7)
*Mycobacteria spp*.	6 (1.7)
Other pathogens	15 (4.4)
Polymicrobial infections	64 (18.6)
No pathogen identified	73 (21.2)

**Therapy**^†^	
Beta-lactams	292 (84.9)
Amoxicilline/Clavulanate	129 (37.5)
Penicilline	54 (15.7)
Flucloxacilline	48 (14.0)
Cephalosporines	33 (9.6)
Piperacilline/Tazobactam	18 (5.2)
Carbapenemes	10 (2.9)
Non-beta-lactams	
Aminoglycosides (used in combination)	10 (2.9)
Fluoroquinolones	8 (2.3)
Antituberculosis drugs	6 (1.7)
Others	28 (8.1)

* seen by an ID during a period of 5 years.

^† ^Antibiotic prescribed for the longest period intravenously during one hospitalization.

^‡ ^Two of 131 (1.53%) *S.aureus *infections were due to methicillin-resistant *S.aureus *(MRSA).

*S. aureus *was the most frequently isolated pathogen, followed by beta-hemolytic and viridans streptococci (Table 3). *S.aureus *was the most frequent pathogen isolated in all categories of infections: endocarditis (76% of cases with a primary pathogen identified), bone and joint infections (58%), primary sepsis (57%), septic thrombosis (56%), skin and soft tissue infections (45%), pneumonia (35%) and in other infections (35%). Polymicrobial infections were observed in 18.6% of cases.

### Therapy and outcome

A beta-lactam antibiotic, most frequently amoxicilline/clavulanate, was used for treatment in 84.9% of the cases (Table 3). The median duration of intravenous and oral antibiotic therapy was 12 days (range 0–117 days) and 1 day (range 0–36 days), respectively.

Treatment according to standard guidelines was possible in 270 (78.5%) of the 344 hospitalizations. A best alternative therapy was prescribed as recommended by an infectious disease specialist for non-compliant patients in 39 (11.3%) cases. Therapy that was not according to an infectious disease specialist advice was prescribed in 35 (10.2%) cases (Figure 1).

For some infections, e.g., for skin and soft tissue infections, sepsis, or pneumonia, 47.1% of the cases were scheduled for additional outpatient therapy after an adequate or best alternative in-hospital course of treatment (Table 4). In 12.2% of the hospitalizations, patients were discharged against medical advice either with or without therapy.

**Table 4:** Discharge data for 344 hospitalizations among 216 IVDU* for treatment of infectious diseases

	**n (%)**
Discharge with oral antibiotics after adequate or best alternative therapy	162 (47.1)
Discharge without antibiotics after adequate or best alternative therapy	117 (34.0)
Discharge against medical advice, with oral antibiotics	29 (8.4)
Discharge against medical advice, without antibiotic therapy	13 (3.8)
Discharge without available follow-up data for antibiotic therapy	1 (0.3)
In-hospital deaths^†^	22 (6.4)
	
Total	344 (100)
	

Readmissions	
< 30 days with relapse of original infection	13 (3.8)
with infection but not relapse of original infection	19 (5.5)
with no infection	15 (4.4)
30–90 days	53 (15.4)
> 90 days	100 (29.1)

Deaths overall (determined from state records for n = 199 patients)	49 (24.6)
After discharge from hospital: not related to treated infection	27 (13.6)
In-hospital (of cases):	
infection related	18 (5.2)
not infection related	4 (1.2)

IVDU = Intravenous drug users.

* seen by an ID during a period of 5 years.

^† ^Described in detail in the lower part of the table.

Complications occurred in 153 hospitalizations (44.5%). Complications noted were acute renal failure (8.4%) and septic shock (3.2%). In 21.5% of the hospitalizations, the patients were admitted to the ICU (median length of ICU stay = 3 days). The presence of pneumonia or endocarditis most often led to ICU admission–36% of all cases of pneumonia and 29.6% of all cases of endocarditis. Non-compliant patient behavior was the most frequent complication (19.8%), and a multiple logistic regression analysis (Table 5) revealed a significant association of non-compliance with psychiatric disorders (OR = 2.4, CI 1.1–5.1, p = 0.03). In hospitalizations of non-compliant patients, the patient was able to follow the prescribed therapeutic program in accordance with standard guidelines in 27.9% of the cases.

**Table 5:** Risk factors associated with patient non-compliance and infection-related mortality (multiple logistic regression analysis) of 344 hospitalizations among 216 IVDU*

**Risk factors for patient's non-compliance**	**OR (95% CI)**	**p-value**
Male gender	1.2 (0.6–2.2)	0.60
Age (per 10 years older)	0.8 (0.5–1.2)	0.26
Alcohol addiction	0.8 (0.4–1.7)	0.55
Psychiatric disorders	2.4 (1.1–5.1)	0.03^†^
HIV infection	0.7 (0.3–1.3)	0.24

**Risk factors for infection-related mortality**	**OR (95% CI)**	**p-value**

Number of infections	1.4 (0.7–2.9)	0.32
Age (per 10 years older)	2.0 (1.0–3.8)	0.04^†^
Male gender	1.0 (0.4–2.6)	0.93
Number of comorbidities	1.0 (0.8–1.3)	0.95

* seen by an ID during a period of 5 years.

^† ^Statistical significance (p < 0.05).

The overall 30-day readmission rate was 13.7% (Table 4). Among readmitted cases, 27.7% had a relapse (overall relapse rate 3.8%). The highest relapse rates occurred with septic thrombosis (5.6%) and endocarditis (3.7%). Both non-compliant patient behavior (OR = 3.7, CI 1.3–10.8, p = 0.02) and non-adherence to treatment guidelines (OR = 3.3, CI 1.1–9.7, p = 0.03) were associated with a significant increase in the relapse rate in univariate analysis. Because of the low number of relapses, no multiple (adjusted) logistic models were performed.

Overall in-hospital mortality was 6.4% (n = 22, Table 4). The highest mortality rates were associated with bacterial endocarditis (13%), pneumonia (12%), and sepsis (9.6%). The age of the patient was the only independent risk factors for infection-related, in-hospital mortality (Table 5). State death record information was available for 199 (92.1%) of the 216 patients, which allowed the evaluation of mortality rates after discharge from the hospital (a total of 590 person-years of follow-up). The median observation time was 35.6 months (range 1.6–73.9 months). A total of 49 (24.6%) patients died during the observation period. Of these 49 patients, 27 (55.1%) died after discharge from the hospital, 3 of them within 30 days (2 for whom death was possibly infection related, 1 who died of a trauma). The remaining 24 deaths occurred more than 30 days after the last date of discharge (median 232 days).

There was no significant difference in non-compliant behavior, relapse- or readmission rate between HIV-infected and HIV-negative patients. The association between infection-related deaths and HIV status during hospitalization was non-significant (OR = 1.9, CI 0.8–4.9, p = 0.161). Overall in hospital mortality (OR = 2.4, CI 1.0–5.6, p = 0.06) and mortality in the follow up period (OR = 2.4, CI 1.3–4.4, p = 0.006) was increased for episodes with HIV-infected patients.

## Discussion

This study investigating 344 hospitalizations in 216 IVDU demonstrates that the vast majority of infectious disease episodes in these patients can be treated successfully by following international guidelines or protocols adapted from these standard guidelines.

Treatment according to guidelines was completed in 78% of hospitalizations, and treatment with a best alternative regimen recommended by a specialist in infectious diseases was possible in an additional 11% of patients who had been identified as non-compliant (Figure 1). Hence, even complex and prolonged intravenous antibiotic treatment can be administered successfully in almost 90% of IVDU. Prescription of alternative treatment programs and non-adherence by patients were associated with a twofold and fourfold increase in the relapse rate, respectively. However, when the recommendations of an infectious disease specialist were followed, relapse occurred in only 2.6% of the cases (Figure 1). Physicians should be aware that deviating from recommended protocols is associated with a higher rate of treatment failure and relapse. Making compromises in treatment strategies cannot be justified on the basis of a belief that a patient may not adhere to the prescribed program. In an earlier study of HIV-infected patients, it was demonstrated that IVDU also are at risk of receiving inadequate treatment for HIV, in part because physicians did not follow the treatment programs that were recommended by infectious disease specialists [27].

The extent of adherence to recommended treatment programs for infections in IVDU and its relationship to treatment outcome had not been analyzed prior to this study. Self-reported, non-adherence to therapy is a problem for IVDU who are being treated for infectious diseases, just as it is for IVDU who are receiving antiretroviral therapy [28]. Therefore, a patient's adherence to a program of taking oral antibiotics after the patient has been discharged from the hospital cannot be taken for granted. The high rate (>90%) of IVDU who were participating in a structured opioid maintenance program may partially explain the high rate of adherence to treatment programs by patients in the study population, even though almost all patients reported using illicit drugs in addition to their official maintenance drug dose, which points to their severe addiction.

Major psychiatric disorders were associated with a reduced adherence in our study, which confirms other reports in the literature that describe the more risky behaviors encountered in these patients [13]. Gender, age, alcohol addiction, and HIV infection had no significant impact on patient compliance in a multivariate analysis (Table 5).

The mortality rate for IVDU is known to be greater than that of the general population [29]. In our study population, in-hospital mortality (6.4%) was rather low (Table 4). Most deaths during the study occurred more than 30 days after discharge from the hospital and were not related to prior hospitalization for treatment of infectious disease. Similar observations were made in a longitudinal study among opioid addicts [29]. The only independent risk factor for in-hospital death in this study was older age (Table 5). In-hospital deaths of IVDU were primarily due to uncontrolled active infections despite their receiving adequate therapy for the infections according to standard guidelines. The highest mortality rate was observed in patients with bacterial endocarditis (13%). This rate is comparable to infection-related, in-hospital mortality rates attributable to endocarditis among in hospital patients who are not addicts (14%; in-house, unpublished data). The data includes mortality due to both right-sided and left-sided and staphylococcal and streptococcal endocarditis. Although IVDU may not seek medical treatment until late in the course of an infectious disease and have a greater number of co-morbidities and complications, they tend to be younger and have a predominant involvement of the tricuspid valve [30]. Both factors contribute to a lower mortality compared to the 20–25% mortality in staphylococcal endocarditis of the left heart [24,31-33].

Our study corroborates results from earlier studies [1,2,34] that show that skin and soft tissue infections are the most frequently occurring infections in IVDU (Table 2). In about half the cases of skin and soft tissue infection, surgical intervention was implemented in addition to antibiotic therapy. The duration of intravenous antibiotic therapy is quite short for skin and soft tissue infections, which may partly explain the high rate of adherence to our guidelines.

The majority (36 of 59) of deep venous thromboses in IVDU was septic, constituting 10.5% of hospitalizations. These results emphasize the importance of screening for signs and symptoms of deep venous thrombosis in IVDU.

In almost 80% of the hospitalizations, the causative pathogen for the infection could be identified. The predominance of *S. aureus *and streptococci (Table 3) confirms earlier results [5,6,31,35-38]. Methicillin-resistant *S. aureus *(MRSA) may lead to outbreaks of infection among IVDU [39,40], although in our study, MRSA was isolated to only two cases–both in the same patient, which confirms the low prevalence of MRSA in our region [41,42]. Amoxicilline/clavulanate was the antibiotic used most frequently to treat infections (Table 3), which is comparable to results in other studies, e.g., a similar study conducted in the emergency department of the University Hospital Basel studying the general patient population [43].

The main limitation of this study is its retrospective character. The source of our data was restricted to hospital medical records and the detailed records of the specialists in the infectious diseases department. A second limitation is that detailed information about the duration of drug addiction in patients was missing in most cases. Therefore, we were not able to substantiate the impression that the risk for hospitalization due to infections increased with the number of years of addiction. Nevertheless, the age of our study population is rather high (median 38 years, mean 37 years) in comparison with the mean age of IVDU in a Swiss heroin maintenance program (mean 20.2–27 years, depending on the addictive drug that was being used, 2001) [44]. Hence, younger patients were underrepresented in hospitalized IVDU in our study. Only in part, this can be explained by the increasing mean age of IVDU over time as shown in the US recently [45]. A third limitation is the fact that co-morbidities could not be investigated in detail, for example, the fact that there was no reference to co-morbidity in a patient's final medical report does not completely exclude the possibility of co-morbidity having existed and we were not able to standardize the assessment of non-compliant behavior. As fourth limitation, we analyzed only hospitalization seen by infectious disease specialists and a generalization to all IVDU hospitalizations may be limited. Nevertheless, we assume that we were consulted for the more difficult situations rather enforcing the good appropriateness.

The strength of this study was the high number of hospitalizations that were investigated in a population of IVDU. Although this study was a retrospective investigation, we were able to extract significant information from the general medical charts and from the detailed reports of the infectious disease specialists by using a sophisticated case report form that included precise definitions of different infectious diseases. Also, our outcomes were validated by the state death records, which confirmed the favorable outcome in most patients.

## Conclusion

Appropriate antibiotic therapy in hospitalized IVDU is generally practicable and successful. In a minority of cases, alternative treatments may be indicated, although they are associated with a higher risk of failure. Complicated disease courses and readmissions are common in IVDU; but in this study, only a small percentage of readmissions were due to relapse. Overall mortality in this population of IVDU is high, but it does not appear to be related to prior hospitalizations for infectious disease.

## Competing interests

The author(s) declare that they have no competing interests.

## Authors' contributions

DM, UF and MB designed the study, supervised the data collection and interpreted the analyses. NV and DM collected and analysed data and drafted the first manuscript. GL and BL acquired data. DM and MW performed the statistical analysis. All authors critically revised the final draft of the manuscript.

## Pre-publication history

The pre-publication history for this paper can be accessed here:


